# Flower scent of *Ceropegia stenantha*: electrophysiological activity and synthesis of novel components

**DOI:** 10.1007/s00359-019-01318-4

**Published:** 2019-03-13

**Authors:** Annemarie Heiduk, Jean-Paul Haenni, Ulrich Meve, Stefan Schulz, Stefan Dötterl

**Affiliations:** 1 Department of Biosciences, Plant Ecology, University of Salzburg, Hellbrunnerstr. 34, 5020 Salzburg, Austria; 2 Department of Plant Systematics, University of Bayreuth, Bayreuth, Germany; 3 Natural History Museum Neuchâtel, Neuchâtel, Switzerland; 4 Institute of Organic Chemistry, Technische Universität Braunschweig, Brunswick, Germany

**Keywords:** Flower scent, Electrophysiology, Scatopsidae, 3-Acetyloxy-4-phenylbutan-2-one, 3-Acetyloxy-1-phenylbutan-2-one

## Abstract

In specialized pollination systems, floral scents are crucial for flower–pollinator communication, but key volatiles that attract pollinators are unknown for most systems. Deceptive *Ceropegia* trap flowers are famous for their elaborate mechanisms to trap flies. Recent studies revealed species-specific floral chemistry suggesting highly specialized mimicry strategies. However, volatiles involved in fly attraction were until now identified in *C. dolichophylla* and *C. sandersonii*, only. We here present data on *C. stenantha* for which flower scent and pollinators were recently described, but volatiles involved in flower–fly communication stayed unknown. We performed electrophysiological measurements with scatopsid fly pollinators (*Coboldia fuscipes*) and identified 12 out of 13 biologically active floral components. Among these volatiles some were never described from any organism but *C. stenantha*. We synthesized these components, tested them on antennae of male and female flies, and confirmed their biological activity. Overall, our data show that half of the volatiles emitted from *C. stenantha* flowers are perceived by male and female fly pollinators and are potentially important for flower–fly communication in this pollination system. Further studies are needed to clarify the role of the electrophysiologically active components in the life of scatopsid fly pollinators, and to fully understand the pollination strategy of *C. stenantha*.

## Introduction

Most angiosperms show mutualistic interactions with their animal pollinators, but 4–6% of flowering plants are pollinated by deceit (Renner 2006). The flowers/inflorescences of deceptive plants cheat their pollinators by either advertising a reward (e.g., breeding substrate, mating site, food) which they do not provide or by providing a reward different from what they advertise (Renner 2006). Thereby, pollinators are fooled by visual, olfactory, or tactile signals which generate a sensory impression of a desired object they will, however, not find in the flowers. In specialized deceptive pollination systems, olfactory signals (i.e., floral scents) are considered most important for pollinator attraction (Vereecken and McNeil 2010; Johnson and Schiestl 2016; Goodrich and Jürgens 2018; Wee et al. 2018); though additional traits, such as morphological or tactile cues, help to keep the disappointed pollinators interested, and trapping devices may even detain them in the flowers to achieve successful pollination (see Araceae: Chartier et al. 2014; Apocynaceae-Asclepiadoideae: Vogel 1961; Heiduk et al. 2017; Aristolochiaceae: Oelschlägel et al. 2009, 2015; Orchidaceae: Singer 2002).

Celebrities among such cheating plants are the highly sophisticated trap flowers of *Ceropegia* L. (Apocynaceae-Asclepiadoideae-Ceropegieae). This species-rich genus (> 220 species) is restricted to Old World tropical and subtropical habitats with hotspots of diversification in South-East Africa, India, Madagascar, and China (Meve and Liede-Schumann 2007; Bruyns et al. 2015). *Ceropegia* species are predominantly visited by small Diptera, though other insects/arthropods enter the flowers as well (Ollerton 1999). Hitherto, no other insects but flies where found to carry pollinaria, thus *Ceropegia* species are functionally highly specialized on Diptera as pollinators. As recently reviewed by Ollerton et al. (2017), 16 fly families are confirmed as *Ceropegia* pollinators plus additional nine families are known visitors of *Ceropegia* flowers. The currently available data (Ollerton et al. 2017) show that most *Ceropegia* species specifically attract flies of only one or two families and genera, respectively, though a few species are visited and pollinated by up to seven fly families (Ollerton et al. 2017). Recent studies on floral chemistry suggest that this pollinator specificity is achieved through species-specific floral scents (Heiduk et al. 2015, 2016, 2017). Data on flower scent are available for a total of 14 *Ceropegia* species (Heiduk et al. 2017) and these data point towards specialized mimicry strategies to attract fly pollinators, such as mimicry of injured/dying insects as a food source for kleptoparasitic flies (Heiduk et al. 2015, 2016), and rotting fruits (Heiduk et al. 2017). However, the compounds which are physiologically and/or behaviorally active in the respective fly pollinators are only identified for two species, i.e., *C. dolichophylla* (Heiduk et al. 2015) and *C. sandersonii* (Heiduk et al. 2016), which are both pollinated by kleptoparasitic milichiid flies. For all other *Ceropegia* species, the key floral volatiles for pollinator attraction remain to be identified. For this purpose, electrophysiological measurements are a powerful tool to screen for compounds that are potentially involved in attraction; however, their attractiveness finally has to be proven by behavioral assays (Heiduk et al. 2015, 2016). Being highly selective chemical lures, *Ceropegia* flowers provide an ideal system to learn more about fly attractive chemicals, and the behavior as well as life history of flies that act as pollinators.

The flowers of *Ceropegia stenantha* K. Schum. are pollinated by Scatopsidae both in native (Tanzanian habitats) and non-native ranges. In the plants’ native habitat, four scatopsid fly species of the three genera *Neorhegmoclemina*, *Rhegmoclemina*, and *Swammerdamella* were described as pollinators (Heiduk et al. 2017). Flowers of plants cultivated in Germany were found to be pollinated by two different scatopsid species of the genera *Coboldia* and *Swammerdamella* (Heiduk et al. 2017).

Scatopsidae are scavengers or detritivores in larval stages (Freeman 1985; Haenni and Vaillant 1994; Haenni 1997) and are known to be associated with rotting organic material, such as fermenting leaf litter (Perez et al. 2013). Scatopsidae are also described as nectar and pollen-feeding flower visitors (Larson et al. 2001; García-Robledo and Mora 2007; Woodcock et al. 2014). One of the non-native pollinator species of *Ceropegia stenantha*, i.e., the oyster mushroom fly *Coboldia fuscipes* (Meigen 1830), is famous for being a pest insect of economic importance. Just as its common name suggests, the oyster mushroom fly causes severe damage to production of edible mushrooms, such as shiitake, champignon, and oyster mushroom (Yi et al. 2015, and references therein). Thus, mushroom-derived chemicals possibly attract these cosmopolitan flies; as their immature stages develop in diverse media, i.e., cow dung (Skidmore 1991), fungi (Dely-Draskovits 1974; 40 species of host fungi), various decaying vegetal (Duda 1928; Lyall 1929; Rabello and Forattini 1962) and dead animal matter (Ayre 1995), volatiles related to these substrates are most likely attractive as well. Though the antennal structure of these flies has been studied in detail (Zhang et al. 2016), nothing is known about the chemical perception in *C. fuscipes*.

To understand why *Coboldia fuscipes* is attracted to flowers of *Ceropegia stenantha*, we tested antennae of this pollinating scatopsid species to floral scent samples. Antennae of male and female flies consistently responded to a total of 13 components. Among them were novel chemical compounds known so far only from flowers of *C. stenantha*. They were synthesized and, together with other electrophysiologically active floral components tested as synthetic standards in further electrophysiological measurements to confirm their biological activity.

## Materials and methods

### Plant material

**Fig. 1:**
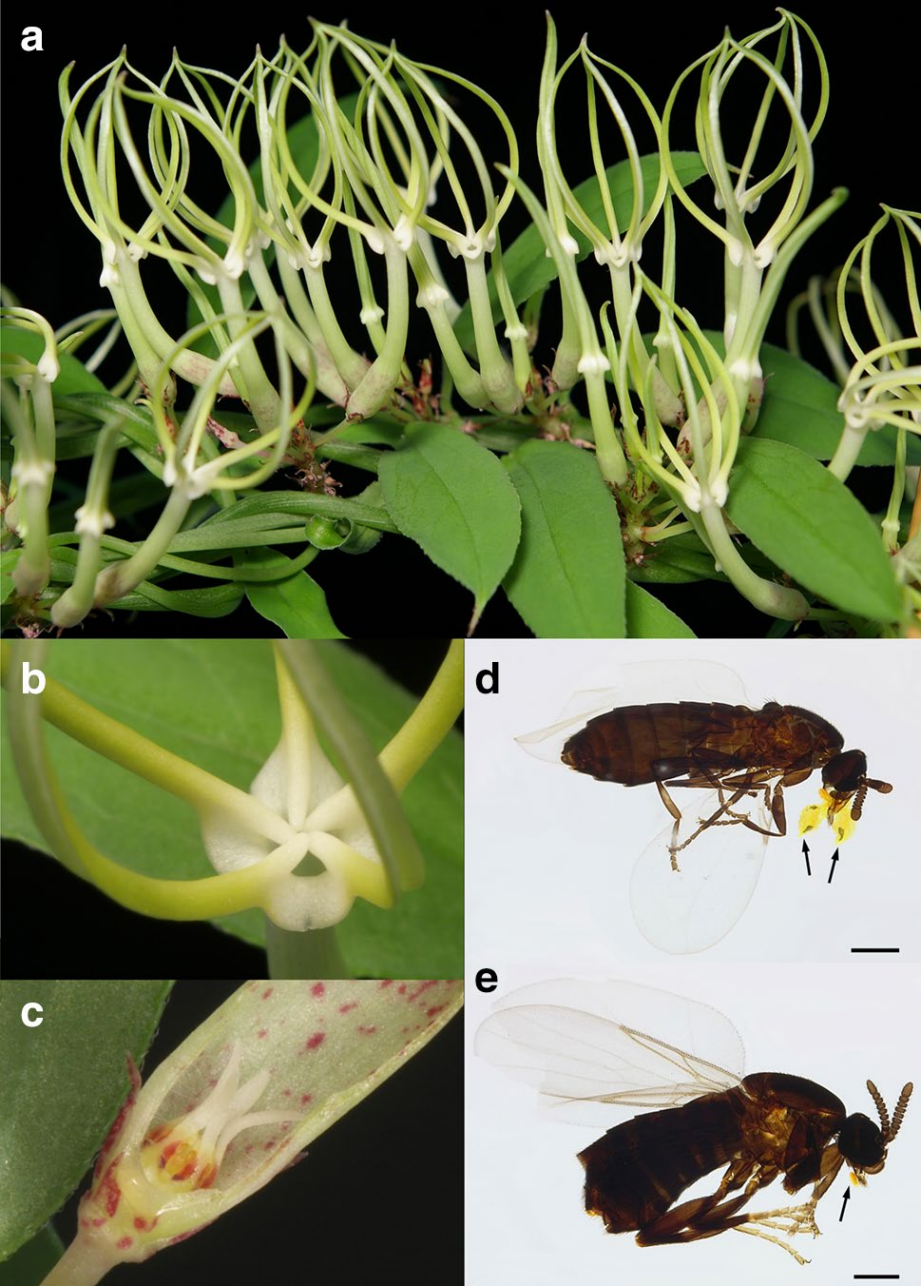
Flowers and pollinators of *Ceropegia stenantha*. Mass displays of simultaneously open flowers (**a**). Entrance to floral tube limited by ligula-like, adaxial extensions of the corolla lobe bases (**b**). Cross-section through the narrow basal inflation showing limited space around the gynostegium (**c**). Two pollinating species of Scatopsidae with pollinaria attached to their proboscises: male of *Swammerdamella brevicornis* (**d**); male of *Coboldia fuscipes* (**d**). Arrows indicate the two pollinia of a single pollinarium (in **d**) and a detached corpuscle (in **e**). Scale bar: 250 µm. Photographs: U. Meve (**a**–**c**) and A. Heiduk (**d, e**)

*Ceropegiastenantha* is a root-, stem- and leaf-succulent twiner with smooth and slender, lanceolate leaves (Meve 2002). This fairly small perennial is scrambling or twining on shrubs, rarely exceeding 1 m in height; typically it flowers in mass displays (Fig. 1a). The corolla (up to 3 cm in length) is faintly greenish-white with small yellowish lobes. The corolla lobe tips, which are apically fused to form a cage-like structure (Fig. 1a), together with the adaxial extensions of the corolla lobe bases limit the access to the tube (Fig. 1b). The corolla tube is only slightly curved, exceptionally narrow, and merges into an also very narrow basal inflation with relatively little space around the gynostegium (Fig. 1c). *C. stenantha* is distributed throughout (sub-)tropical eastern Africa from Sudan to South Africa, where it grows in grassland areas (savanna) and open woodlands (see Masinde 2004).

For this study, *Ceropegia stenantha* was grown from seeds collected in Tanzania (Senga, Rukwa Region; leg. R. von Blittersdorf, 12. April 2011) and cultivated in greenhouses at the University of Bayreuth (UBT), Germany, in 2012. Some plants set flowers (vouchers held at UBT) and, though far from its natural habitat, were visited and successfully pollinated by scatopsid flies, i.e., *Swammerdamella brevicornis* (Meigen, 1830; Fig. 1d) and *Coboldia fuscipes* (Meigen, 1830; Fig. 1e) (Heiduk et al. 2017).

### Volatile collection

Two types of flower scent samples, i.e., thermal desorption (TD) and solvent acetone (SAc), were collected from *Ceropegia stenantha* flowers using dynamic headspace methods (Dötterl et al. 2005). For collection of TD samples, three flowers (first day of anthesis) of one plant individual were enclosed singly in polyester oven bags (Toppits®, Germany) for 10 min. Accumulated volatiles were subsequently pulled from the bag through a small adsorbent tube filled with a mixture of 1.5 mg Tenax-TA (mesh 60–80) and 1.5 mg Carbotrap B (mesh 20–40) (both Supelco, Bellefonte, PA, USA; Heiduk et al. 2015) for 5 min using a membrane pump (G12/01EB, Rietschle Thomas Inc., Puchheim, Germany) with the flow rate adjusted to 200 ml/min. An ambient air sample was taken in a similar way and used as a control to specify floral volatiles in the samples taken from flowers. For collection of the SAc sample, the same three individual flowers were sampled for 4 h each at a flow rate of 100 ml/min using bigger adsorbent tubes filled with 15 mg Tenax-TA and 15 mg Carbotrap B (Heiduk et al. 2015). These tubes were then eluted with 60 µl of acetone (SupraSolv, Merck KgaA, Germany) each, and the three elutes were pooled to obtain a single SAc for electrophysiological measurements and chemical synthesis (see below).

The TD samples were already used in a comparative study on *Ceropegia* flower scent (Heiduk et al. 2017) and re-analyzed in the present study for comparison to the SAc sample. We here show semi-quantitative data obtained by re-analyzing the TD samples.

### Chemical analyses (GC/MS)

To identify electrophysiologically active components (see below), 1.0 µl of the SAc sample was analyzed by gas chromatography/mass spectrometry (GC/MS) using a Shimadzu GCMS-QP2010 Ultra equipped with an AOC-20i auto injector (Shimadzu, Tokyo, Japan) and a Zebron ZB-5 fused silica column (5% phenyl polysiloxane; length: 30 m, inner diameter: 0.32 mm, film thickness: 0.25 µm, Phenomenex). One µl of the sample was injected (injection temperature: 220 °C; split ratio 1:1) and the column flow (carrier gas: helium) was set at 3 ml/min. The GC oven temperature was held at 40 °C for 1 min, then increased by 10 °C/min to 220 °C and held for 2 min. The MS interface worked at 220 °C. Mass spectra were taken at 70 eV (in electron ionization mode) from mass-to-charge ratio (*m*/*z*) 30 to 350, and data were processed using the GCMSolution package, Version 2.72 (Shimadzu Corporation 2012). Flower scent components of the GC/MS spectra were identified using the mass spectral data bases (for details see Heiduk et al. 2017).

### Synthesis of novel chemical components

**Fig. 2:**
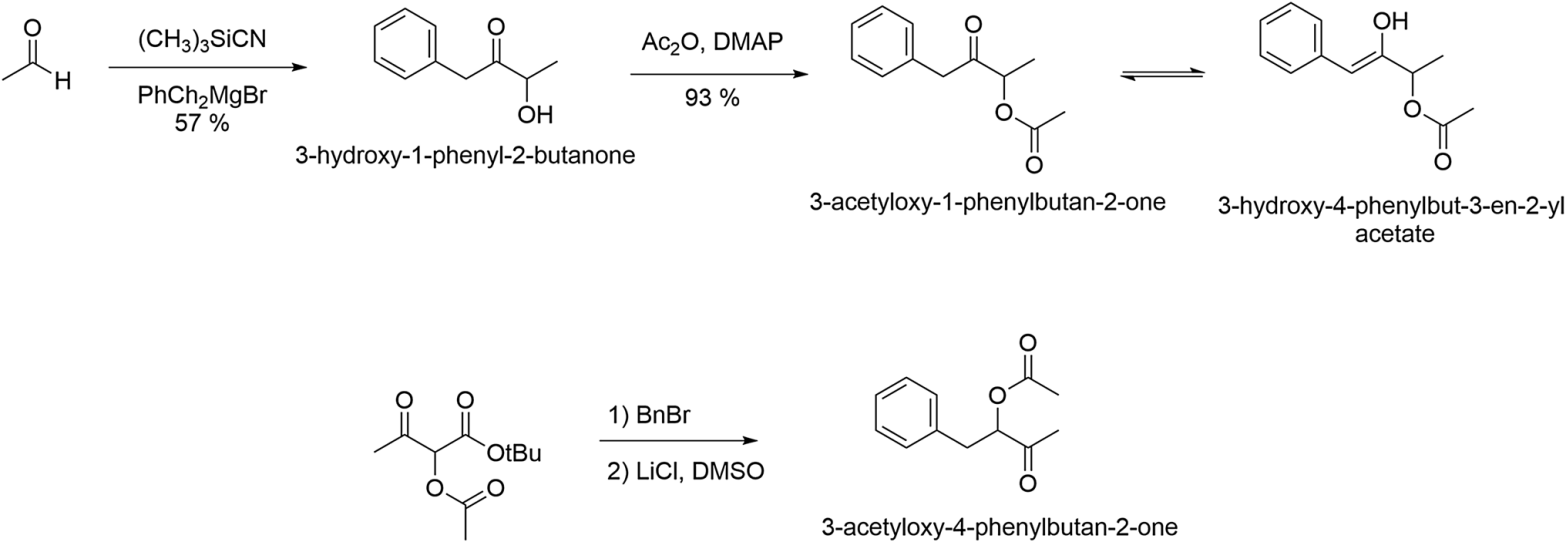
Synthesis of 3-acetyloxy-1-phenylbutan-2-one and 3-acetyloxy-4-phenylbutan-2-one

3-Hydroxy-1-phenyl-2-butanone was prepared in 57% yield according to the procedure of Guo et al. (1999) from acetaldehyde, trimethylsilylcyanide and benzylmagnesium bromide. Acetylation with acetanhydride in the presence of *N,N*-dimethylaminopyridine (DMAP) furnished 3-acetyloxy-1-phenylbutan-2-one in 93% yield. The compound was isolated as a mixture of tautomers as shown in Fig. 2 (^1^H NMR (400 MHz, CDCl_3_) δ: 1.36 (d, 3H), 1.46 (d, 3H), 2.07 (s, 3H), 2.12 (t, 3H), 3.79 (m, 2H), 5.17 (q, 1H), 5.56 (q, 1H), 6.34 (s, 1H), 7.17–7.40 (m, 5H). 3-Acetyloxy-1-phenylbutan-2-one: ^13^C NMR (100 MHz, CDCl_3_) δ: 16.2, 20.7, 45.6, 74.3, 127.1, 128.6, 129.5, 133.0, 170.3, 205.1. 3-Hydroxy-4-phenylbut-3-en-2-yl acetate: ^13^C NMR (100 MHz, CDCl_3_) δ: 17.9, 20.9, 70.1, 118.7, 127.9, 128.5, 128.6, 146.3, 168.2, 170.0.).

The isomer 3-acetyloxy-4-phenylbutan-2-one was synthesized according to the method developed by Scheid et al. (2004) by alkylation of *tert*.-butyl 2-acetyloxyacetoacetate (Fig. 2, ^1^H NMR (400 MHz, CDCl_3_) δ: 2.07 (s, 3H), 2.08 (s, 3H), 3.00 (dd, 2H), 3.10 (dd, 1H), 5.20 (dd, 1H), 7.19–7.34 (m, 5H). ^13^C NMR (100 MHz, CDCl_3_) δ: 20.6, 26.8, 36.7, 79.0, 127.0, 128.5, 129.3, 135.8, 170.3, 205.3).

### Electrophysiological measurements (GC/EAD)

The SAc sample of *Ceropegia stenantha* flower scent and a mixture of eleven synthetic floral components (purity: 90–99%, equal volumes, diluted in acetone, final concentration: 10^− 3^; v/v) were tested on antennae of male and female *Coboldia fuscipes* flies (Scatopsidae). The mixture contained benzyl alcohol, benzaldehyde, 2-phenylethanol, phenylacetaldehyde, 1,4-dimethoxybenzene, 2-methoxyphenol, 1-phenyl-1,2-propandione, 3-hydroxy-1-phenylbutan-2-one, 1-phenyl-2,3-butandione, 3-acetyloxy-1-phenylbutan-2-one, and 3-acetyloxy-4-phenylbutan-2-one. The synthetic standard compounds were purchased from Sigma-Aldrich, available in the collection of Stefan Schulz, or de novo synthesized (see above).

A total of 22 runs were performed with seven male and seven female fly individuals (one antenna per insect). Five male and three female antennae were tested to both the floral SAc sample and the synthetic mixture, three female antennae and one male antenna were tested only to floral scent, and one antenna of both sexes was tested only to the synthetic mixture.

Measurements were performed with an Agilent 7890A (Santa Clara, California, USA) gas chromatograph. The GC was equipped with a flame ionization detector (FID), an EAD setup (heated transfer line, 2-channel USB acquisition controller; provided by Syntech; Kirchzarten, Germany), and a Zebron ZB-5 column (5% phenyl polysiloxane; length: 30 m, inner diameter: 0.32 mm, film thickness: 0.25 µm, Phenomenex). For each run, 1.0 µl of the sample was injected in splitless mode (injector temperature: 250 °C; oven temperature: 40 °C). The split opened 30 s after injection, and the oven heated by 10 °C/min up to 220 °C. The column was split at the end by a µFlow splitter (Gerstel, Mühlheim, Germany; nitrogen was used as make-up gas) into two deactivated capillaries, one (length: 2 m, inner diameter: 0.15 mm) leading to the FID setup, the other (length: 1 m, inner diameter: 0.2 mm) leading to the EAD setup. The outlet of the EAD was placed in a cleaned, humidified air flow directed over the fly antenna.

Flies used for measurements were anesthetized (CO_2_) and their heads cut off. Two glass micropipette electrodes were filled with insect Ringer’s solution (8.0 g/l NaCl, 0.4 g/l KCl, 4.0 g/l CaCl_2_) and connected to silver wires. The caudal side of the head was connected to the reference electrode, and the recording electrode was placed in contact with the last flagellomere. The very tip of the flagellomere had to be cut because antennae were densely covered with mitrotrichiae which hampered immersion of the antennae into the Ringer’s solution.

## Results

### Chemical analyses (GC/MS)

The re-analyses of *Ceropegia stenantha* flower scent samples (Heiduk et al. 2017) added further 4 components to the 19 components previously detected (Heiduk et al. 2017). Thereof, 1,4-dimethoxybenzene and two unknown compounds (KRI 1460, KRI 1470; see Table 1) were found in only trace amounts in the TD samples, but in higher amounts in the SAc sample. A third unknown compound was only present in the SAc sample (see below). From the total of 23 components, 12 were identified and verified through authentic standards (see Table 1, Heiduk et al. 2017). All identified components were aromatic compounds which in total contributed an average of 96% to the total amount of scent. Two compounds, i.e., benzaldehyde (64.6 ± 14.9%) and 1-phenyl-2,3-butanedione (22.8 ± 11.6%) were by far more abundant than all other components and on average accounted to 87.5 ± 3.3%. Due to keto-enol tautomerism, 1-phenyl-2,3-butanedione appeared with two peaks at two different retention times in the chromatograms (1-phenyl-2,3-butanedione: KRI 1213, 3-hydroxy-4-phenylbut-3-en-2-one: KRI 1444) and the relative amount contributed by 1-phenyl-2,3-butanedione was calculated as the sum of both peaks. Only four further compounds contributed > 1% to the total amount of scent released: an unknown component (KRI 1508; 2.5 ± 0.67%), 1,2-dimethoxybenzene (1.1 ± 0.9%), 3-acetyloxy-4-phenylbutan-2-one (2.8 ± 0.76%), and 3-acetyloxy-1-phenylbutan-2-one (2.3 ± 0.64%). The latter two components were determined as novel chemical compounds (see Heiduk et al. 2017) and synthesized as standards for electrophysiological measurements in the present work (see below). The mean total amount of scent emitted per flower was 13.6 ± 2.1 ng/min.

**Table 1:** Floral volatiles identified in *Ceropegia stenantha* after having re-analyzed scent samples already analyzed by (Heiduk et al. 2017). 1,4-Dimethoxybenzene and the two unknown components KRI 1460 and KRI 1470 were not detected in the previous analysis. The total amount of scent emitted from flowers as well as the electrophysiological activity of floral volatiles in male and female *Coboldia fuscipes* (Scatopsidae) flies is also reported. KRI: Kovats retention index; EAD: electroantennographic detection; tr: trace amount < 0.05

	KRI	*Ceropegia stenantha* flower scent [%]			EAD response of *Coboldia fuscipes*
		Flower A	Flower B	Flower C	
Total amount trapped per flower [ng/min]		15.7	10.7	14.4	#Female/#male
Aromatic components		97.2	94.2	96.4	(*n* = 7 each)
Benzaldehyde^S^	963	**84.3**	**48.3**	**61.4**	7/6
Benzyl alcohol^S^	1035	0.7	0.9	0.5	7/7
Phenylacetaldehyde^S^	1050	0.1	0.1	0.1	6/7
2-Methoxyphenol^S^	1094	0.1	0.3	0.2	6/7
2-Phenylethanol^S^	1118	0.3	0.4	0.3	7/7
1,2-Dimethoxybenzene^S^	1146	0.2	0.7	2.4	4/5
1,4-Dimethoxybenzene^S^	1167	tr	tr	tr	6/4
1-Phenyl-1,2-propandione^S^	1173	0.4	0.5	1.1	7/7
1-Phenyl-2,3-butanedione^S^	1213	**6.5**	**29.1**	**20.1**	7/6
3-Hydroxy-1-phenyl-2-butanone^S^	1356	0.1	0.6	0.1	6/6
3-Hydroxy-4-phenylbut-3-en-2-one*^S^	1444	1.2	**7.0**	4.5	7/6
3-Acetyloxy-4-phenylbutan-2-one^S,a^	1475	1.8	3.5	3.2	7/7
3-Acetyloxy-1-phenylbutan-2-one^S,a^	1499	1.4	2.9	2.6	7/7

Unknown components^*n* = 10^		2.8	5.8	3.6	
*m/z*: 43, 65, 88, 91, 119, 146, 164	1460	tr	tr	tr	
*m/z*: 43, 65, 78, 91, 119, 146, 164	1470	tr	tr	tr	

Numbers in bold: whenever total amounts of individual compounds are > 5%

^S^compound verified through authentic standard

^a^compound synthesized in present work for having an authentic standard available

*tautomeric form of 1-phenyl-2,3-butanedione

### Identification of novel chemical components

**Fig. 3:**
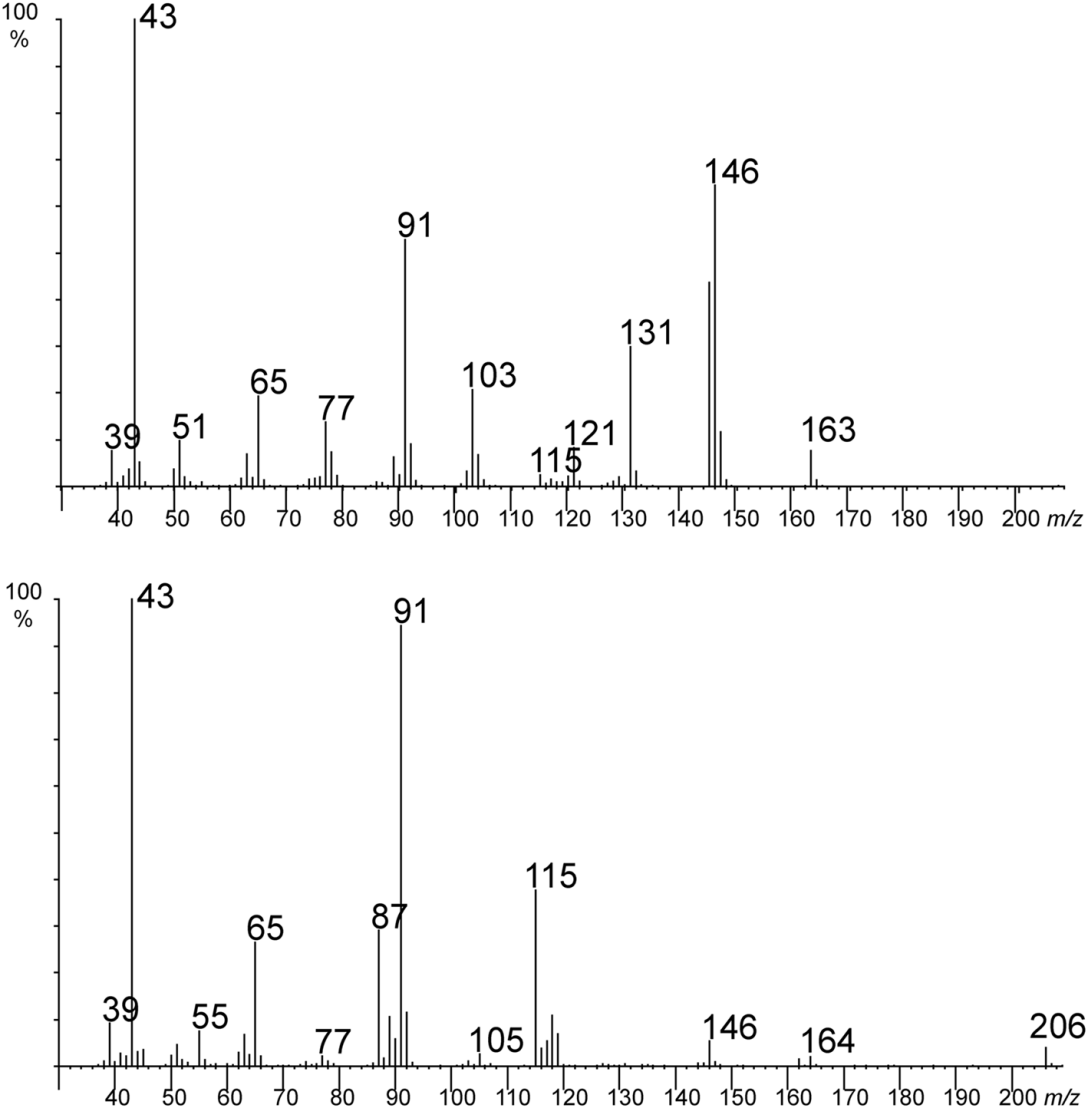
Mass spectra of 3-acetyloxy-4-phenylbutan-2-one (upper) and 3-acetyloxy-1-phenylbutan-2-one (lower)

The structures of the two compounds 3-acetyloxy-4-phenylbutan-2-one and 3-acetyloxy-1-phenylbutan-2-one, previously not reported as natural products, were deduced from their mass spectra (Fig. 3). The spectrum of the latter indicates a benzyl group by the ion *m*/*z* 91 and at least one acetyl-group (*m*/*z* 43). The molecular ion at *m*/*z* 206 loses 42 or 60 amu fragments, consistent with an acetyloxy-group. An acetyloxy-group at C-2 of the chain leads to an ion at *m*/*z* 87, and the related ion *m*/*z* 115 points to a neighboring carbonyl group. Connecting these fragments leaves 3-acetyloxy-1-phenylbutan-2-one as the sole likely structure.

The second compound showed similarities, such as *m*/*z* 91 (benzyl group) and 146 (loss of acetic acid), with the first spectrum. Although the molecular ion is missing, the loss of 43 amu indicates a methylketone fragment. We therefore proposed 3-acetyloxy-4-phenylbutan-2-one as the structure for this compound. Both compounds are acylated acetoins, which easily undergo internal redox reaction resulting in switching of the positions of the keto- and hydroxy-groups. Therefore, the co-occurrence of both compounds is very often observed in nature.

These structural assignments were confirmed by co-injection of floral samples and authentic material obtained by synthesis as described in the experimental section.

### Electrophysiological measurements (GC/EAD)

**Fig. 4:**
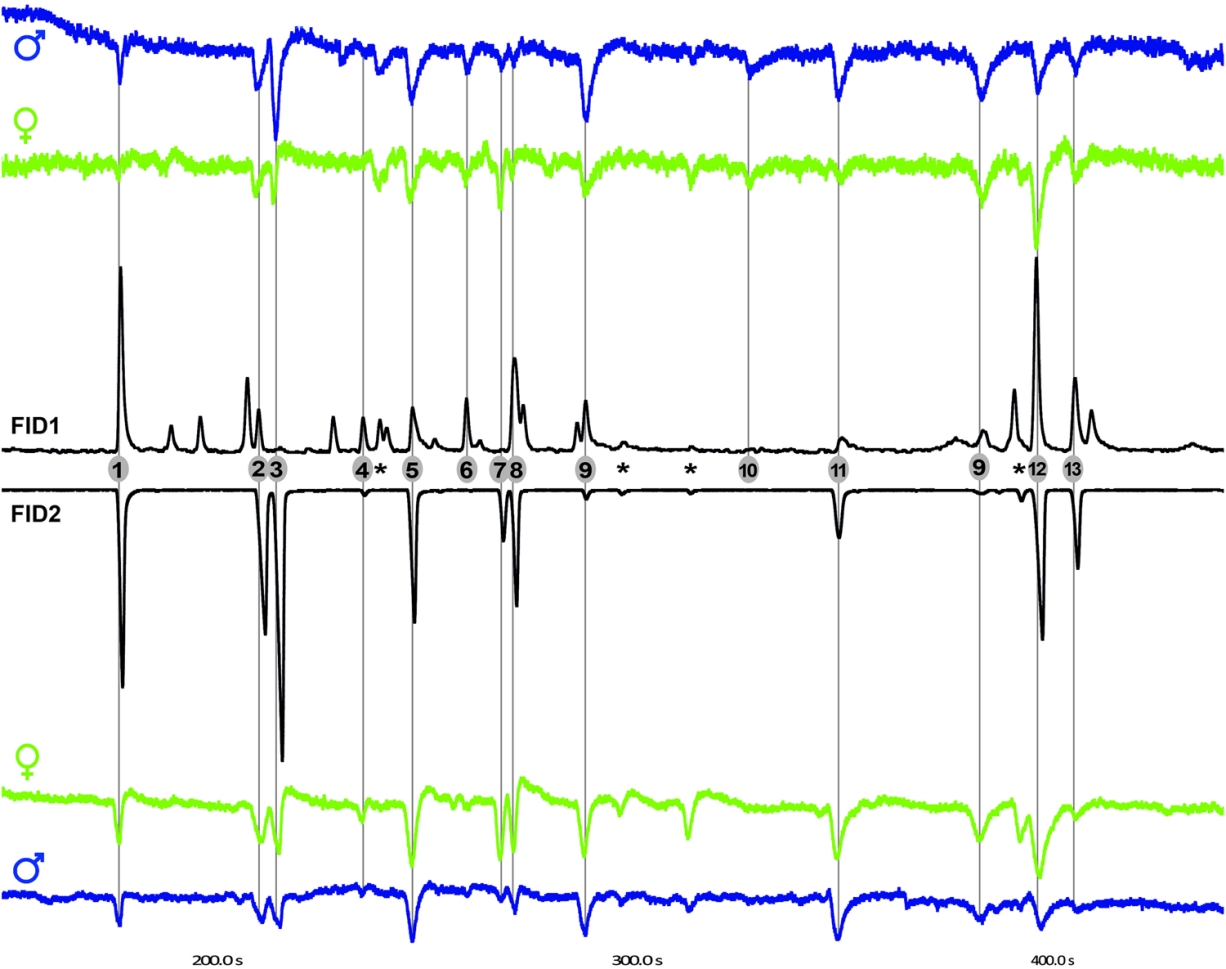
Antennal responses of a female (green) and a male (blue) *Coboldia fuscipes* (Scatopsidae) fly to a natural flower scent sample of *Ceropegia stenantha* (FID1) and a mixture of 11 synthetic substances identified from the natural sample (FID2). 1, benzaldehyde; 2, benzyl alcohol; 3, phenylacetaldehyde; 4, 2-methoxyphenol; 5, 2-phenylethanol; 6, 1,2-dimethoxybenzene; 7, 1,4-dimethoxybenzene; 8, 1-phenyl-1,2-propandione; 9, 1-phenyl-2,3-butanedione/3-hydroxy-4-phenylbut-3-en-2-one; 10, unknown component not detected in TD samples; 11, 3-hydroxy-1-phenyl-2-butanone; 12, 3-acetyloxy-4-phenylbutan-2-one; 13, 3-acetyloxy-1-phenylbutan-2-one. Asterisk response to contamination

Both male and female *Coboldia fuscipes* flies gave obvious antennal signals to a total of 13 floral scent components, 11 of which were verified through authentic standards (see Table 1; Fig. 4). All 14 (7 females, 7 males) tested flies responded to benzyl alcohol, 2-phenylethanol, 1-phenyl-1,2-propandione, 3-acetyloxy-4-phenylbutan-2-one, and 3-acetyloxy-1-phenylbutan-2-one. Antennae of 13 flies responded to benzaldehyde, phenylacetaldehyde, 2-methoxyphenol, and 1-phenyl-2,3-butanedione. 3-Hydroxy-1-phenyl-2-butanone was active in 12 flies, and 1,2- and 1,4-dimethoxybenzene were active in nine and ten flies, respectively. Furthermore, a total of six flies responded to an unknown component only present in the SAc sample in amounts too low for identification (see Fig. 4, #10). All components (except for the unknown compound #10) elicited antennal responses in at least 64% of tested flies and were consistently active in repeated measurements with single fly antennae.

## Discussion

*Ceropegia stenantha* flowers were found to emit a floral scent composed of 23 components of which 19 were already detected by Heiduk et al. (2017). Thereof, 12 aromatic components were identified and verified through authentic standards, for some of which the synthesis is described in the present paper. In electroantennographic measurements, 13 out of 23 floral scent components were consistently perceived by male and female *Coboldia fuscipes* flies (Fig. 4; Table 1). Among these compounds were 3-acetyloxy-4-phenylbutan-2-one and 3-acetyloxy-1-phenylbutan-2-one, which are not known from another organism than *C. stenantha* (see also Heiduk et al. 2017).

### Fly pollinators and floral scent chemistry

*Ceropegia stenantha* flowers are exclusively visited and pollinated by Scatopsidae, and we assume that this highly specific attraction is achieved by floral chemistry. Chemical attraction of fly pollinators is assumed for *Ceropegia* species in general (Vogel 1961; Heiduk et al. 2017) but was thus far only confirmed in two species (Heiduk et al. 2015, 2016). Our electrophysiological measurements with *Coboldia fuscipes* (Fig. 4), which is one out of six scatopsid species identified as *C. stenantha* pollinators (Heiduk et al. 2017), point towards an important role of floral volatiles in pollinator attraction. As suggested for *Ceropegia* species in general (Vogel 1961), we assume that *C. stenantha* has a deceptive pollination strategy and it might either mimic a food source, oviposition site, or pheromones (see Vogel 1961; Heiduk et al. 2017).

Deduced from the chemical nature of most floral compounds identified in *C. stenantha*, we doubt that they chemically resemble female oviposition sites. The chemical profile of C. *stenantha* does not fit to what is described as typical oviposition site mimicry (Jürgens et al. 2013) and the aromatic-based floral scent is rather sweet smelling, whereas oviposition site mimics generally emit unpleasant scents. Furthermore, the high abundance of male individuals present inside the flowers (see above) is again intriguing in this regard; although it is described in other flies that males are attracted to oviposition sites for mating purpose (see Maier and Waldbauer 1979; Papaj 1994; Bonduriansky and Brooks 1999).

### Occurrence and signaling of biologically active volatiles of *Ceropegia stenantha* in other plants

Several of the floral components perceived by *C. fuscipes* are known from rewarding flowering plants and listed among the most widespread floral volatiles (Knudsen et al. 2006; El-Sayed 2018). Some components thereof were found to be biologically active in different insects. The main scent component benzaldehyde, and the less abundant components benzyl alcohol and 2-phenylethanol are known from up to 60% of angiosperm families (Knudsen et al. 2006) and are attractive to moths (Bruce and Cork 2001; Landolt et al. 2013; Sun et al. 2016), mosquitoes (Jhumur et al. 2008), hemipterans (Fontan et al. 2002), and honey bees (Mas et al. 2018). Phenylacetaldehyde is known from 30 plant families (Knudsen et al. 2006), and described to be an honest reward indicator for honey and bumble bees (Knauer and Schiestl 2015; Mas et al. 2018). 1,2- and 1,4-dimethoxybenzene are less common but still found in floral scents of 14 different plant families (Knudsen et al. 2006) and are attractive to, for example, leaf beetles (Ventura et al. 2001). Rather seldom described as flower volatile is 2-methoxyphenol; however, in different combinations with the above-mentioned components, it can be found in floral scents of moth pollinated night-flowering plants (Knudsen and Tollsten 1993; Jürgens et al. 2002; Dötterl and Jürgens 2005). Interestingly, the second most abundant component found in *C. stenantha*, 1-phenyl-2,3-butanedione, has only been identified in floral scents of very few plants (Joulain 1987; Wong and Teng 1994; Gross et al. 2016). Likewise, 1-phenyl-1,2-propandione is rarely described as floral volatile; it occurs, for example, in the nectar-rewarding orchid *Gymnadenia odoratissima* in combination with benzaldehyde, benzyl alcohol, phenylacetaldehyde, and 1-phenyl-2,3-butanedione which were all found to be perceived by pollinating Lepidoptera (Huber et al. 2005).

### Mimicry strategy of *Ceropegia stenantha*

The occurrence and bioactivity of above mentioned components suggests that they signal a food source (e.g., nectar, pollen) to attract pollinators. Flowers of *C. stenantha* do not offer such rewards but may attract food-seeking Scatopsidae to its trap flowers by emitting reward-indicating aromatic components commonly found in rewarding flowers. Scatopsidae are known to visit such flowers to feed on pollen and/or nectar (Apiaceae: Haenni 1997; Asteraceae: Haenni 1990; Honěk et al. 2016; Dipsacaceae: Haenni 1984; Rubiaceae: García-Robledo and Mora 2007) and probably use chemical cues to find them. Food deception through signals that indicate a reward/rewarding species is a well-studied strategy with different facets, e.g., Batesian mimicry and generalized food deception (see Johnson and Schiestl 2016). However, generalized deception as well as mimicry of nectar and pollen mainly relies on visual cues (for review see Johnson and Schiestl 2016). Though *C. stenantha* flowers might contrast well against the background and thus be salient for flies (Fig. 1a, b), they do not seem to visually resemble a rewarding flower of another plant species. If *C. stenantha* would mimic a food source by emitting likely reward related volatiles, it is still suspicious that only Scatopsidae are found inside the trap flowers.

Scatopsidae often aggregate by hundreds of individuals (male and female) at specific sites not in relation with larval development sites (Fritz 1984; Haenni 2002; Nielsen 2009); however, mating behavior is frequent in these aggregations. The mechanisms triggering this aggregation behavior are unknown. *C. stenantha* flowers attract male and female Scatopsidae of different species (Heiduk et al. 2017); resolving the chemistry behind the attractiveness of the flowers might help to understand the aggregation behavior in Scatopsidae. It has been shown, for example, in Drosophilidae that males and females aggregate using pheromone components which are attractive to both sexes and not necessarily species-specific (Bartelt et al. 1986). Likewise, sexual behavior in *Drosophila* flies is connected to activation of food-sensing neurons which helps males to find females (Grosjean et al. 2011). These neurons are activated by phenylacetaldehyde, a compound also found in *C. stenantha* flower scent. Under the assumption that Scatopsidae have a similar neuronal circuitry, we could hypothesize that *C. stenantha* uses a mixed strategy: emission of aromatic components that are related to a food source (see above) in combination with the novel components which might have a pheromone function. Solvent extracts (dichlormethane, hexane, pentane) as well as TD samples of male and female *Coboldia fuscipes* did not contain 3-acetyloxy-4-phenylbutan-2-one and 3-acetyloxy-1-phenylbutan-2-one, nor any of the other components identified in *C. stenantha* (Heiduk, unpublished data). However, *C. fuscipes* is only 1 of 13 scatopsid fly species attracted to *C. stenantha* flowers and not described from the plant’s native habitat. To exclude sexual mimicry as pollination strategy in *C. stenantha*, extracts of pollinating scatopsid species, especially from the plant’s native range, need to be analyzed for the presence of the components discussed here.

### Outlook

To clarify which of the electrophysiologically active components presented in this paper finally attract the flies to *C stenantha* flowers, field bioassays with different mixtures of synthetic components are needed. As shown for *C. dolichophylla* and *C. sandersonii*, only small subsets of the electrophysiologically active components may successfully attract the flies (Heiduk et al. 2015, 2016). As already suggested by Heiduk et al. (2017), the selective attractiveness of Scatopsidae to *C. stenantha* might be achieved through the novel flower compounds 3-acetyloxy-4-phenylbutan-2-one and 3-acetyloxy-1-phenylbutan-2-one. Our electroantennographic measurements with scatopsid flies now supported this assumption and also indicated the importance of other scent components in this pollination system. Further studies will reveal which of the electrophysiologically active floral components attract the flies. Once the specifically attractive components are identified, their role in the life of scatopsid flies needs to be clarified to understand which sensation floral scent of *C. stenantha* creates in the scatopsid flies.

## Abbreviations

CDCl_3_: Deuterated chloroform
FID: Flame ionization detector
GC/EAD: Coupled gas chromatography/electroantennographic detection
GC/MS: Gas chromatography/mass spectrometry
KRI: Kovats retention index
*m/z*: Mass-to-charge ratio
NMR: Nuclear magnetic resonance
SAc: Solvent acetone
TD: Thermal desorption
UBT: Herbarium of the University of Bayreuth
